# Supplementary material for: Unsupervised Analysis Reveals the Involvement of Key Immune Response Genes and the Matrisome in Resistance to BRAF and MEK Inhibitors in Melanoma
Source: Cancers (Basel). 2024 Jun 24;16(13):2313. doi: 10.3390/cancers16132313 (PMC11240363; doi:10.3390/cancers16132313)
Supplement: Supplementary file 1 [file cancers-16-02313-s001.zip › cancers-3078490-supplementary.pdf]

Supplemental Figure and Tables

Figure S1: Venn Diagram for shared genes in the two DEG sets.

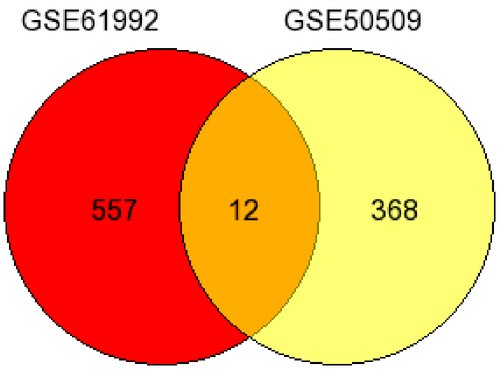

Table S1: Details about patients and tumors.

	Patient	untreated (N=28)	vemurafenib (N=8)	dabrafenib (N=25)	Overall (N=61)
GSE50509					
	Patient 1	1 (3.6%)	0 (0%)	1 (4.0%)	2 (3.3%)
	Patient 2	1 (3.6%)	0 (0%)	1 (4.0%)	2 (3.3%)
	Patient 3	1 (3.6%)	0 (0%)	2 (8.0%)	3 (4.9%)
	Patient 4	1 (3.6%)	0 (0%)	1 (4.0%)	2 (3.3%)
	Patient 5	1 (3.6%)	0 (0%)	2 (8.0%)	3 (4.9%)
	Patient 7	2 (7.1%)	0 (0%)	1 (4.0%)	3 (4.9%)
	Patient 8	2 (7.1%)	0 (0%)	2 (8.0%)	4 (6.6%)
	Patient 9	1 (3.6%)	0 (0%)	1 (4.0%)	2 (3.3%)
	Patient 10	2 (7.1%)	0 (0%)	3 (12.0%)	5 (8.2%)
	Patient 11	2 (7.1%)	0 (0%)	4 (16.0%)	6 (9.8%)
	Patient 12	1 (3.6%)	0 (0%)	1 (4.0%)	2 (3.3%)
	Patient 13	2 (7.1%)	0 (0%)	1 (4.0%)	3 (4.9%)
	Patient 14	1 (3.6%)	0 (0%)	1 (4.0%)	2 (3.3%)
	Patient 17	1 (3.6%)	0 (0%)	1 (4.0%)	2 (3.3%)
	Patient 18	2 (7.1%)	0 (0%)	2 (8.0%)	4 (6.6%)
	Patient 21	1 (3.6%)	0 (0%)	1 (4.0%)	2 (3.3%)
	Patient 23	1 (3.6%)	1 (12.5%)	0 (0%)	2 (3.3%)
	Patient 24	2 (7.1%)	1 (12.5%)	0 (0%)	3 (4.9%)
	Patient 25	1 (3.6%)	1 (12.5%)	0 (0%)	2 (3.3%)
	Patient 28	1 (3.6%)	4 (50.0%)	0 (0%)	5 (8.2%)
	Patient 30	1 (3.6%)	1 (12.5%)	0 (0%)	2 (3.3%)
GSE61992					

Patient	untreated (N=12)	EDT (N=3)	progression (N=11)	Overall (N=26)
Patient 1	2 (16.7%)	1 (33.3%)	1 (9.1%)	4 (15.4%)
Patient 2	1 (8.3%)	0 (0%)	2 (18.2%)	3 (11.5%)
Patient 3	2 (16.7%)	0 (0%)	1 (9.1%)	3 (11.5%)
Patient 4	1 (8.3%)	0 (0%)	1 (9.1%)	2 (7.7%)
Patient 5	1 (8.3%)	0 (0%)	1 (9.1%)	2 (7.7%)
Patient 6	1 (8.3%)	0 (0%)	1 (9.1%)	2 (7.7%)
Patient 7	1 (8.3%)	0 (0%)	2 (18.2%)	3 (11.5%)
Patient 9	1 (8.3%)	0 (0%)	1 (9.1%)	2 (7.7%)
Patient 11	0 (0%)	1 (33.3%)	0 (0%)	1 (3.8%)
Patient 12	0 (0%)	1 (33.3%)	0 (0%)	1 (3.8%)
Patient 10	2 (16.7%)	0 (0%)	1 (9.1%)	3 (11.5%)

Table S2: Significant genes in GSE50509.

SYMBOL	logFC	AveExpr	P.Value
TYRP1	-1.397	10.210	0.046
HSD17B1	-1.133	6.323	0.014
TMEM145	-0.937	7.110	0.001
CAMK1G	-0.903	6.527	0.020
CNFN	-0.890	6.207	0.012
JSRP1	-0.881	5.075	0.024
IRX6	-0.881	7.363	0.022
PRODH	-0.868	5.065	0.033
CKB	-0.867	7.058	0.020
SLAMF9	-0.821	5.875	0.027
F12	-0.809	7.008	0.017
STAG3	-0.797	6.073	0.000
RND2	-0.797	6.304	0.016
PNMA3	-0.796	7.705	0.030
TP53TG1	-0.774	6.690	0.001
KCNIP3	-0.773	5.717	0.010
TPCN2	-0.765	8.283	0.023
TMT1B	-0.758	6.052	0.021
H2AC18	-0.757	8.292	0.004
H2AC19	-0.757	8.292	0.004
AQP11	-0.756	6.190	0.004
KCNS1	-0.754	5.655	0.040
HOXA11-AS	-0.735	6.173	0.039
CASC15	-0.730	7.695	0.011
HS3ST2	-0.728	5.237	0.027

REEP6	-0.726	6.994	0.026
CEBPA	-0.716	7.632	0.026
CHST13	-0.693	5.823	0.010
FOLR2	-0.692	7.320	0.012
H1-9P	-0.690	5.403	0.004
CHST6	-0.685	6.264	0.007
BLVRB	-0.674	8.409	0.007
TSPAN33	-0.654	7.918	0.012
CIB2	-0.653	6.551	0.009
RAP1GAP	-0.650	8.553	0.021
RPP25	-0.649	7.866	0.033
KRT75	-0.641	4.636	0.006
LRFN3	-0.637	8.014	0.023
H4C5	-0.634	5.538	0.000
LIN7B	-0.630	6.134	0.012
NCCRP1	-0.630	4.948	0.048
PAFAH1B3	-0.629	9.349	0.004
SLC2A8	-0.626	8.334	0.002
POPDC3	-0.621	5.391	0.030
H2AC20	-0.615	8.208	0.010
IGLL3P	-0.614	5.977	0.028
OGDHL	-0.610	4.672	0.009
CCL18	-0.609	5.705	0.048
LILRA2	-0.595	5.591	0.017
ZNF296	-0.587	6.460	0.026
H4C8	-0.586	6.541	0.031
PRG4	0.585	6.963	0.030
TPR	0.585	6.963	0.030
GK5	0.586	5.954	0.040
KIAA1217	0.586	5.045	0.037
BRCC3	0.586	5.528	0.047
TLK1	0.587	6.571	0.046
REEP5	0.587	9.403	0.038
PPFIBP1	0.587	6.607	0.002
PLK4	0.587	6.773	0.036
ARGLU1	0.587	10.155	0.034
SNX4	0.588	7.987	0.038
ZNF106	0.589	10.464	0.037
HECTD1	0.590	9.185	0.014
TOM1L1	0.592	5.237	0.017
YEATS4	0.592	8.082	0.043
SLC30A7	0.593	8.849	0.045
RBM12B	0.593	7.361	0.008

UBXN4	0.595	8.914	0.039
ZC3H15	0.595	8.030	0.049
ELF1	0.596	9.166	0.030
CTNNAL1	0.597	7.693	0.014
ZNF700	0.598	7.085	0.016
NT5DC1	0.599	6.353	0.025
MYCBP2	0.599	8.106	0.017
GULP1	0.599	5.101	0.049
MTX3	0.600	7.447	0.039
MRPS31	0.600	8.378	0.034
CLK4	0.601	6.143	0.043
RAD21	0.601	8.748	0.009
SPTY2D1	0.601	6.072	0.011
PDGFD	0.602	6.025	0.031
SNX13	0.603	7.005	0.045
MEIS3P1	0.603	8.090	0.037
ELF2	0.603	5.979	0.000
BST1	0.604	5.296	0.010
KRR1	0.604	5.345	0.023
SH3KBP1	0.605	8.028	0.005
ANO6	0.605	7.931	0.008
CDC42EP3	0.605	5.489	0.029
ARHGAP18	0.606	6.468	0.027
IREB2	0.607	5.780	0.023
TRIM38	0.607	7.141	0.033
LEO1	0.609	5.587	0.022
FNDC3A	0.611	6.470	0.043
PDS5A	0.612	7.821	0.017
UBXN2B	0.612	6.590	0.011
BBX	0.612	8.892	0.001
IFIT2	0.613	7.740	0.045
RB1	0.613	6.120	0.024
CLPX	0.613	7.290	0.029
DYNLT3	0.613	5.481	0.012
LPL	0.613	6.924	0.031
USPL1	0.614	7.342	0.044
ADSS2	0.614	8.720	0.035
ZNF800	0.616	7.077	0.031
MBNL2	0.616	7.339	0.029
TGDS	0.617	7.991	0.012
ARID4B	0.617	6.191	0.013
B4GALT6	0.617	6.092	0.021
TRIM24	0.617	5.647	0.002

UNC5CL	0.618	5.565	0.025
MATR3	0.618	6.876	0.002
SNHG4	0.618	6.876	0.002
ST3GAL5	0.618	9.014	0.005
ZCCHC14	0.619	7.935	0.019
NUP54	0.619	7.838	0.029
PLEKHA3	0.619	5.669	0.013
UGCG	0.619	9.043	0.006
ATOSA	0.620	6.911	0.034
WBP4	0.620	7.561	0.040
ANKRD12	0.620	7.111	0.033
ARMCX5	0.620	7.183	0.012
SFRP4	0.621	5.712	0.043
AASDHPPT	0.623	7.707	0.024
KLHL20	0.625	6.805	0.040
GOLGA8A	0.625	6.989	0.027
GOLGA8B	0.625	6.989	0.027
TBL1XR1	0.625	6.445	0.027
CD46	0.625	7.643	0.048
MTIF3	0.628	8.520	0.027
ZMYM4	0.629	7.549	0.042
VPS26A	0.630	9.786	0.006
RBM26	0.630	6.904	0.043
PPP1R9A	0.630	5.236	0.024
BTBD1	0.632	7.627	0.007
DDX21	0.632	8.866	0.016
NCKAP1	0.633	6.638	0.030
ABCB10	0.633	7.351	0.035
MARCHF7	0.634	9.064	0.034
ZNF217	0.636	7.367	0.040
ROCK1	0.637	5.532	0.007
FNDC3B	0.638	8.561	0.034
SNRK	0.639	8.777	0.006
DMTF1	0.640	7.736	0.047
HIF1A	0.640	6.708	0.038
ADGRL4	0.640	5.533	0.029
TNIK	0.640	4.689	0.006
VPS4B	0.641	8.968	0.005
FRMD6	0.641	6.859	0.016
PCNP	0.641	10.079	0.020
LYRM7	0.641	7.137	0.049
BAZ1A	0.643	6.933	0.010
DDX60L	0.643	5.309	0.014

RPF2	0.643	9.304	0.049
CEP350	0.643	8.643	0.024
ACTR2	0.644	10.023	0.019
DNAJC24	0.644	6.643	0.031
TMEM123	0.644	9.328	0.015
STAMBPL1	0.645	8.397	0.019
PIK3C2A	0.646	6.191	0.037
NFXL1	0.647	6.769	0.026
VAMP7	0.647	8.982	0.042
SOS1	0.647	5.084	0.001
ZBED5	0.647	8.142	0.029
MYO1B	0.648	8.301	0.014
ETV5	0.648	9.458	0.010
HSP90B1	0.648	11.276	0.045
HNRNPH2	0.648	6.751	0.028
RPL36A-HNRNPH2	0.648	6.751	0.028
CD44	0.648	9.561	0.012
TARS1	0.648	9.177	0.047
KRCC1	0.649	7.395	0.038
AP1S2	0.649	11.534	0.030
SORBS2	0.649	5.307	0.001
DOCK11	0.651	7.271	0.022
EP300	0.652	7.599	0.004
TMX4	0.653	8.645	0.005
ZNF277	0.653	7.787	0.039
ORC3	0.653	7.439	0.045
HLTF	0.654	7.115	0.050
PUM3	0.654	8.257	0.043
GPBP1	0.655	7.677	0.038
WDR26	0.658	8.001	0.022
DDX60	0.658	6.209	0.015
ZC3H7A	0.659	7.628	0.012
ZEB2	0.660	7.994	0.039
BCLAF1	0.662	7.257	0.015
LYAR	0.663	8.055	0.024
CCT8	0.663	10.655	0.015
COG6	0.663	7.129	0.032
PRRX1	0.663	6.681	0.002
UPF3A	0.666	7.729	0.009
ZFR	0.666	8.841	0.046
PIP4P2	0.666	7.544	0.030
EXOC1	0.666	6.478	0.002

GNAQ	0.667	6.099	0.007
IPO7	0.668	5.595	0.020
CAB39	0.669	9.763	0.011
NPHP3	0.673	7.315	0.045
ABCC4	0.676	6.038	0.050
USP33	0.676	6.357	0.000
OSGIN2	0.676	6.254	0.014
TANC1	0.676	8.292	0.025
ANKRD49	0.678	6.770	0.008
CFAP97	0.678	6.022	0.018
CDK6	0.679	8.536	0.035
NMD3	0.680	7.995	0.044
PGM2	0.681	7.441	0.021
TUSC3	0.682	6.929	0.048
MBLAC2	0.682	6.319	0.028
TAX1BP1	0.683	10.055	0.006
HSD17B12	0.684	9.639	0.023
TCEA1	0.684	6.873	0.004
SPIRE1	0.685	9.892	0.010
PRSS23	0.688	8.797	0.020
CD164	0.688	7.528	0.021
SECISBP2L	0.689	7.782	0.006
NKTR	0.691	7.388	0.026
SUCLA2	0.691	7.427	0.027
SSH1	0.691	6.437	0.003
LEF1	0.693	9.777	0.005
TRPC1	0.694	5.537	0.014
TRMT13	0.695	6.616	0.041
GPR180	0.697	7.959	0.037
TCF4	0.697	6.511	0.050
EMCN	0.698	5.354	0.027
RDX	0.698	8.005	0.029
IQGAP1	0.699	8.970	0.001
MYO5C	0.699	5.968	0.010
ANLN	0.700	7.351	0.048
ZNF12	0.700	6.851	0.044
ITPRID2	0.701	6.414	0.026
RAB11FIP2	0.702	7.183	0.035
ANKRD50	0.704	7.377	0.032
LTN1	0.705	6.410	0.013
ARRDC4	0.705	6.425	0.015
PAFAH1B1	0.706	8.891	0.008
DLGAP5	0.709	7.109	0.028

RAB5A	0.710	7.675	0.024
SASH1	0.711	8.302	0.012
ITGA1	0.713	5.194	0.032
ACAP2	0.714	6.476	0.019
EPB41L3	0.714	8.450	0.016
LXN	0.714	8.228	0.018
TUT7	0.714	6.024	0.021
GMCL1	0.715	6.806	0.004
ATAD2	0.716	6.811	0.019
CPNE8	0.716	7.704	0.039
SPATA18	0.717	4.881	0.017
ARID5B	0.718	9.552	0.003
BIVM	0.719	8.057	0.019
CAMK2N1	0.719	8.210	0.015
IL1RAP	0.720	5.856	0.003
ZRANB2	0.721	8.427	0.013
MICU2	0.721	8.811	0.042
CALD1	0.723	8.139	0.010
ZNF770	0.723	7.317	0.025
NSA2	0.725	10.013	0.018
NPAT	0.725	5.620	0.005
FAP	0.725	6.291	0.043
ANKDD1A	0.726	7.438	0.020
RBM34	0.728	8.376	0.043
HTR2B	0.730	5.342	0.008
CDH19	0.730	5.518	0.002
CENPU	0.732	6.460	0.016
KCTD3	0.732	8.476	0.041
ATP1B1	0.732	8.813	0.034
NDC80	0.733	6.819	0.019
PCM1	0.734	8.399	0.044
THOC1	0.735	7.580	0.035
FNBP4	0.735	9.154	0.019
SPRY2	0.739	10.490	0.004
CDC5L	0.740	7.660	0.036
RSRC1	0.741	6.263	0.032
PHACTR2	0.744	6.697	0.016
ATAD1	0.746	7.903	0.013
CCSER2	0.751	6.965	0.022
CBR4	0.752	7.177	0.025
PCSK1	0.754	4.953	0.019
CPEB4	0.754	6.422	0.014
FAM114A1	0.758	5.303	0.003

HNRNPH3	0.758	8.100	0.046
EIF3J	0.759	7.583	0.016
SLC25A36	0.759	7.314	0.020
BMPR2	0.759	8.247	0.009
HDGFL3	0.766	7.605	0.023
SCOC	0.766	8.433	0.020
PPWD1	0.768	6.754	0.019
STC1	0.770	7.088	0.047
SLC2A3	0.770	10.561	0.009
YTHDC2	0.772	5.519	0.007
IFI44	0.775	8.217	0.039
SDCBP	0.775	10.943	0.024
RIOK2	0.775	7.149	0.044
IFIH1	0.776	6.981	0.041
NAMPT	0.780	7.095	0.035
MTM1	0.783	5.788	0.006
RHOBTB3	0.784	9.619	0.014
PTGR3	0.788	7.364	0.004
ZNF25	0.788	6.776	0.020
OLR1	0.789	6.562	0.011
EIF2A	0.791	8.812	0.032
ARID2	0.792	6.225	0.003
PLXNC1	0.793	6.374	0.023
OSBPL8	0.795	7.637	0.006
PKD2	0.798	7.961	0.004
AMD1	0.801	8.589	0.004
RND3	0.804	7.411	0.039
TMX3	0.807	6.906	0.008
STRN3	0.815	7.495	0.022
TBC1D4	0.816	7.459	0.010
PCMTD1	0.816	8.127	0.033
U2SURP	0.818	6.869	0.016
ACSL3	0.821	7.624	0.002
SCUBE2	0.822	5.241	0.037
SPDL1	0.823	6.396	0.027
CAP2	0.823	8.010	0.045
MPDZ	0.828	7.633	0.001
ANXA1	0.831	10.611	0.014
PELI2	0.831	6.588	0.015
DAAM1	0.831	7.271	0.012
TRAPPC8	0.831	8.139	0.012
TDRD3	0.832	6.857	0.029
PLOD2	0.833	7.326	0.009

SPATA13	0.834	5.790	0.004
C1GALT1	0.836	7.162	0.009
SAMD9	0.837	6.909	0.017
CEACAM1	0.838	8.093	0.036
REV3L	0.843	5.714	0.006
MEF2C	0.848	6.489	0.004
SULF1	0.851	7.457	0.030
KAT2B	0.857	8.983	0.004
LARP7	0.860	7.138	0.018
SPP1	0.862	10.658	0.022
SGCE	0.868	9.236	0.012
TBC1D23	0.876	6.200	0.002
NFE2L2	0.876	8.873	0.038
CXCL8	0.879	8.472	0.034
TYW3	0.879	6.691	0.007
FAM3C	0.881	7.031	0.022
BIVM-ERCC5	0.885	7.967	0.014
ERCC5	0.885	7.967	0.014
SKIC3	0.885	8.543	0.015
HNRNPA2B1	0.886	9.128	0.013
ITPR1	0.886	6.693	0.014
JAK1	0.895	6.922	0.004
SRFBP1	0.895	6.555	0.006
PTPRZ1	0.904	6.842	0.030
FZD6	0.909	7.146	0.028
CSGALNACT1	0.927	6.469	0.001
CGNL1	0.928	6.317	0.023
ABCE1	0.932	8.137	0.012
IFI16	0.936	9.699	0.008
UBL3	0.952	9.965	0.008
PTPN13	0.954	6.146	0.006
SH3D19	0.968	7.073	0.001
PNN	0.980	7.813	0.041
PDE3B	0.981	6.063	0.009
STXBP3	0.989	6.729	0.014
GSTM3	0.995	6.541	0.004
PI16	1.012	5.920	0.024
PHIP	1.013	8.125	0.038
BAALC	1.019	5.937	0.007
KLF9	1.053	8.250	0.001
NT5C3A	1.062	9.182	0.001
CYP4V2	1.066	8.356	0.003
DUSP6	1.070	8.620	0.001

SPARCL1	1.097	8.347	0.016
POSTN	1.102	8.301	0.045
BCHE	1.119	7.780	0.027
GNG12	1.140	7.019	0.003
MCOLN3	1.159	7.696	0.001
ITGAV	1.160	8.403	0.009
PLA2G2A	1.185	5.187	0.002
TSPAN7	1.196	8.390	0.001
ALDH1A3	1.231	8.493	0.022
OLIG1	1.275	6.429	0.009
SHISA2	1.377	8.212	0.014
SNX10	1.421	8.792	0.002

Table S3: Significant genes in GSE61992

SYMBOL	logFC	AveExpr	P.Value
March1	-0.64964	8.4453	0.00486
March4	-0.73249	7.64454	0.024609
A2M	-0.59769	6.81293	0.016698
ABCA6	-0.77774	6.84812	0.008135
ABLIM2	-0.61676	6.55757	0.019498
ACAP1	0.620014	5.26997	0.029464
ADAM23	-0.72175	4.3864	0.031886
ADAMDEC1	-0.62983	6.46988	0.011114
ADAMTS9	0.701443	4.9401	0.028643
ADAMTSL2	1.70309	4.7983	0.012215
ADAP2	0.58874	4.75598	0.020671
ADCK2	-1.01316	6.31463	0.001706
ADHFE1	-0.62531	8.48693	0.008446
ADORA2B	1.37976	4.912	0.008714
AGK	-0.98324	4.30844	0.021838
AHNAK2	1.06169	7.11285	0.049142
ALDH1B1	0.607779	8.29243	0.003754
ALDH2	-0.6982	4.77649	0.025027
ALPK1	-0.72727	8.9454	0.033041
AMICA1	0.705342	7.64007	0.023151
ANGPTL4	-0.99892	7.64499	0.003544
ANGPTL7	0.611059	4.43945	0.030113
ANKRD37	-1.40023	6.78512	0.018953
AOAH	0.796959	6.95129	0.010014
APCDD1L	0.662032	8.48848	0.00611
APOLD1	-1.14865	10.1386	0.01284
ARHGAP18	0.700369	5.35743	0.02484

ARHGAP9	-1.00271	6.82981	0.001843
ARHGDIB	-0.71855	7.51381	0.004669
ATAD3A	0.983873	6.04883	0.015156
ATP8B4	-0.67162	6.52469	0.02363
AURKA	-1.04072	11.0096	0.004848
AURKB	-0.91469	5.8132	0.005246
B3GALT4	-0.60468	7.35188	0.006421
BARX2	-0.82111	5.76685	0.007946
BATF	-0.92745	5.72927	0.002566
BEGAIN	-0.80909	6.55723	0.002577
BEX5	-1.07384	5.10776	0.02908
BMP2	-0.76965	4.7908	0.01256
BNIP3	-0.87291	4.91569	0.01314
BOC	1.46136	5.1272	0.028545
BRAF	-1.64374	8.9298	0.000112
BST2	-1.14865	10.1386	0.01284
BTK	-0.9558	5.93245	0.001529
BTN2A2	0.599232	8.80695	0.018535
BTN3A1	-1.74116	8.74859	0.005695
BTN3A3	-0.94912	6.20821	0.04411
BTNL9	-0.61857	6.7222	0.021913
C2	-0.87738	5.29374	0.01017
C2CD2	-1.64374	8.9298	0.000112
CA12	2.72083	6.03521	0.011935
CA5B	-0.77924	6.04051	0.003253
CA9	-0.92934	8.68538	0.013145
CACNA1H	-1.54805	6.5582	0.003065
CADPS2	-0.73444	7.69532	0.005285
CASP1	0.880538	4.8539	0.013558
CCL2	-0.63826	4.32932	0.011816
CCL5	-0.61857	6.7222	0.021913
CCNF	-0.61857	6.7222	0.021913
CCR7	-0.60748	5.29377	0.010645
CCT6B	-0.91184	6.83401	0.007669
CD2	-0.61559	4.39252	0.006924
CD247	1.33057	6.08171	0.004323
CD27	-0.93423	7.06045	0.00597
CD300LF	-0.70632	11.0852	0.034156
CD37	0.771769	5.91615	0.024171
CD3D	-0.8057	6.39887	0.01304
CD3E	-0.73966	7.06401	0.015339
CD4	0.738903	7.31336	0.013443
CD48	-0.72175	4.3864	0.031886

CD52	0.892758	8.56111	0.039001
CD53	-0.61703	5.06347	0.001549
CD55	0.718453	8.16199	0.027612
CD6	0.607573	6.51908	0.016913
CD7	-0.73894	6.86556	0.025017
CD74	0.658284	8.67557	0.014727
CD8A	-0.62504	4.67356	0.020073
CDC20	-0.96904	9.26879	0.00172
CDC42EP2	-1.13499	9.00514	0.000159
CDCA4	1.35045	7.88217	0.005199
CDCA5	1.35045	7.88217	0.005199
CDCA8	-0.73957	11.0369	0.009793
CEBPA	0.933129	8.39379	0.010892
CERCAM	0.797065	12.2197	0.018189
CHCHD10	-0.6213	6.39319	0.015438
CHPF	-0.73268	5.00619	0.014085
CHRNA1	0.706199	7.40179	0.02731
CILP	-0.9863	6.05668	0.012843
CKAP2L	-0.63148	5.12001	0.018441
CKB	0.925435	4.38634	0.003128
CKS2	-0.83717	9.64688	0.018899
CLECL1	-0.6613	5.03551	0.023607
CMTM5	0.631343	6.08013	0.022093
CNTN1	-0.9558	5.93245	0.001529
COMTD1	-0.94942	8.02858	0.014809
CORO1A	-0.65871	7.42619	0.010607
CORO2A	0.832975	3.97345	0.016851
CRIP2	-0.61559	4.39252	0.006924
CSF1R	0.644833	6.09392	0.005364
CSNK1D	2.72083	6.03521	0.011935
CTSO	1.06957	7.61771	0.02161
CTSS	0.592306	4.85509	0.01609
CTSW	0.591196	4.52508	0.02545
CXCL10	0.754366	6.19596	0.024911
CXCL5	0.892267	4.64996	0.018512
CXCL9	-0.66589	5.37409	0.016458
CXCR6	-0.99892	7.64499	0.003544
CYP2U1	-1.20719	7.53032	0.02475
CYR61	1.10017	10.2399	0.038567
CYTH4	0.924239	8.64356	0.048162
DAB1	1.18905	8.64322	0.019596
DBP	-0.8082	7.30261	0.010733
DCBLD1	-0.89281	5.90618	0.006947

DDAH1	-0.76691	8.46622	0.002534
DDIT4	-0.92869	4.91372	0.001475
DEFB103B	0.768769	5.55322	0.00528
DENND2D	-0.75112	5.57315	0.009043
DHX58	0.620323	5.81793	0.00468
DLK1	-1.40979	6.75682	0.000377
DNAJC15	0.729928	8.58631	0.030023
DNER	0.901942	7.702	0.024874
DOCK2	0.809859	6.42787	0.027427
DOCK8	-0.6509	7.12586	0.024426
DOCK9	-1.08411	7.95732	0.021926
DOK2	0.700369	5.35743	0.02484
DPEP2	-1.09758	9.97148	0.006008
DPYD	-0.67745	7.21724	0.026219
DSP	-1.0724	7.7157	0.000555
DUSP1	-0.79303	9.61496	0.023455
DUSP4	0.620014	5.26997	0.029464
EAF2	-0.61083	10.5243	0.009642
ECHDC3	-0.59201	4.61735	0.032502
EFNB2	-0.65901	7.5528	0.008636
EIF4EBP1	0.854399	7.25797	0.021154
EME1	0.805608	4.48396	0.018145
EMP3	-0.91509	4.99978	0.020841
ENPP2	-0.6541	4.01958	0.023442
ENPP6	1.15883	10.1921	0.003953
ENSA	-0.92779	6.09004	0.007941
EOMES	0.658612	9.44373	0.020113
EPHA4	-1.16605	5.01232	0.003143
EPSTI1	0.916941	9.14644	0.045219
ERC2	0.824532	7.13253	0.021349
ESPN	-1.07384	5.10776	0.02908
FAM113B	-0.72416	4.86992	0.010187
FAM118A	-0.6741	5.97566	0.029617
FAM13B	-1.33883	8.60468	0.049715
FAM26F	-0.64265	4.88066	0.032344
FAM27A	-1.03538	7.20467	0.04943
FAM46C	-1.05168	9.14415	0.010379
FAM57A	-0.86526	4.07566	0.0033
FAM64A	0.905197	4.22419	0.013992
FAM83D	0.692505	4.63808	0.023484
FBP1	0.737	6.77388	0.013972
FBXL16	-0.61797	5.59769	0.007245
FBXL21	-0.59097	6.43895	0.0112

FCGRT	-1.11985	11.2701	0.008771
FCHO2	1.08019	6.39055	0.003059
FCN1	-1.19528	8.7608	0.002497
FES	-0.95452	5.16781	0.006733
FEZ1	0.880538	4.8539	0.013558
FGD3	0.768204	5.70411	0.021856
FGF11	-0.63826	4.32932	0.011816
FLI1	-1.29285	5.64592	0.001895
FLJ22536	-1.03538	7.20467	0.04943
FLT3LG	0.963423	5.72666	0.033147
FMO1	-0.73444	7.69532	0.005285
FMO4	-1.12101	7.57496	0.004552
FOLR2	-0.63617	5.70956	0.016868
FOXC1	-0.7653	6.35834	0.014963
FOXM1	0.933129	8.39379	0.010892
FREM1	-0.78385	4.44206	0.010665
FRG1	-1.11304	6.31977	0.01445
FRMD3	-0.84851	5.43968	0.007546
FTHL3	1.53806	5.04678	0.037185
GAS2L1	-0.9863	6.05668	0.012843
GATA5	-1.2507	6.70385	0.020015
GATS	-0.78129	6.24215	0.007726
GBP2	-1.08411	7.95732	0.021926
GBP3	0.627779	4.56868	0.013187
GBP5	-0.69657	7.43573	0.034234
GEM	-0.80867	6.13516	0.004438
GGTLC2	-0.75556	6.29134	0.046664
GIMAP1	-0.97712	5.44051	0.019371
GIMAP2	0.662032	8.48848	0.00611
GIMAP4	0.770445	4.3042	0.017301
GIMAP5	0.83023	3.67981	0.021529
GIMAP8	-0.90056	7.28611	0.017124
GINS4	1.3667	3.71522	0.029345
GK	0.738903	7.31336	0.013443
GLI2	0.880538	4.8539	0.013558
GLI4	-0.6613	5.03551	0.023607
GNLY	-1.08994	5.69189	0.00951
GPC3	1.12488	7.59864	0.031659
GPNMB	2.00287	4.73142	0.027875
GPR160	-1.02791	8.26894	0.001556
GPR65	1.48662	4.85594	0.014469
GPSM3	-0.61703	5.06347	0.001549
GPT2	0.713222	4.63281	0.005223

GSG2	-1.77278	6.52486	0.009769
GSPT2	0.905197	4.22419	0.013992
GVIN1	-0.92745	5.72927	0.002566
GZMA	-1.06259	5.61933	0.023276
GZMB	1.50673	5.59679	0.033999
GZMH	-0.73268	5.00619	0.014085
GZMK	-0.86494	7.25905	0.010059
H3F3B	-0.83535	7.536	0.006952
HAPLN1	-1.28608	6.69466	0.004711
HAVCR2	0.902523	6.22055	0.006805
HCLS1	0.717257	8.21068	0.013545
HCP5	-2.03251	7.70816	0.028457
HCST	0.748092	6.13797	0.007855
HERC6	-0.93583	5.0857	0.000502
HES2	0.796959	6.95129	0.010014
HIPK2	0.632859	6.63868	0.005434
HIST1H4C	1.3658	5.61043	0.032801
HJURP	-0.7125	6.71017	0.008642
HK2	-1.14865	10.1386	0.01284
HK3	-0.93072	7.43418	0.003053
HKDC1	0.892267	4.64996	0.018512
HLA-B	0.700834	4.69033	0.023103
HLA-DMA	-0.89281	5.90618	0.006947
HLA-DMB	0.786335	6.96546	0.020283
HLA-DOA	0.66592	4.44266	0.001273
HLA-DPA1	-0.9745	4.54572	0.021762
HLA-DQA1	1.33911	5.04228	0.029542
HLA-DRA	-0.62483	6.04644	0.006275
HLA-DRB6	-0.81877	5.50996	0.032074
HLA-E	-0.91509	4.99978	0.020841
HLA-F	0.58874	4.75598	0.020671
HLA-G	-1.35108	6.01275	0.000855
HLA-H	-1.2907	6.01575	0.002031
HSH2D	0.683497	5.18864	0.024367
HSPA2	-0.61729	5.49279	0.020965
HYAL3	-0.79277	9.383	0.002349
ICOS	-0.74887	6.23168	0.00291
IFI35	-0.66589	5.37409	0.016458
IFI44	1.36136	5.98017	0.019992
IFI6	1.00563	7.40784	0.00543
IFIT1	1.35451	9.84318	0.007557
IFIT5	1.13071	5.27131	0.00705
IFITM1	2.00287	4.73142	0.027875

IFNG	-1.17796	6.15403	0.038877
IKZF1	-1.2502	6.26544	0.004316
IL10RA	-1.15096	6.2246	0.000363
IL18RAP	1.70309	4.7983	0.012215
IL2RB	0.768769	5.55322	0.00528
IL32	-0.83697	9.76507	0.022527
IL4I1	-0.7629	4.53461	0.001368
IL6	1.18704	9.43782	0.007781
IL6R	-1.24735	6.5858	0.007004
INPP5D	0.665814	6.78138	0.016318
IPPK	0.755085	7.06778	0.014272
IRF1	-0.7467	10.3766	0.009419
IRF7	-0.75194	6.57045	0.003553
IRF8	-0.71718	5.28882	0.002107
IRF9	-0.6555	6.19145	0.016632
ITGAL	0.704375	9.71078	0.017988
ITGB2	-1.43638	5.94954	0.00045
ITGB3	0.73937	7.60201	0.014359
ITPRIP	0.701443	4.9401	0.028643
JAG1	-0.91766	8.67437	0.006541
JHDM1D	0.60397	6.10217	0.048605
JPH1	0.902523	6.22055	0.006805
KBTBD10	-0.60468	7.35188	0.006421
KCNE4	-0.8835	10.3061	0.008569
KCNF1	1.34353	4.98175	0.003101
KCNK1	1.25454	6.45834	0.011613
KIF4A	1.11091	6.45649	0.027417
KIR2DL3	1.86792	5.18742	0.002298
KISS1R	-0.77924	6.04051	0.003253
KLHL6	0.608575	8.75638	0.021287
KLRB1	0.706692	7.03957	0.033059
KLRD1	1.0866	10.1617	0.027085
LAG3	0.611059	4.43945	0.030113
LAIR2	-0.72824	5.5079	0.029326
LANCL2	-0.62874	5.76002	0.00941
LAX1	1.1097	4.84815	0.021644
LCE1D	0.914716	5.97495	0.011563
LCP1	-1.02791	8.26894	0.001556
LDLR	0.769656	7.8014	0.031773
LEF1	-1.02895	6.09587	0.004813
LETM1	-0.73722	5.12061	0.010034
LGALS9	0.933129	8.39379	0.010892
LMNB2	-0.62874	5.76002	0.00941

LOC154761	0.924239	8.64356	0.048162
LOC400759	0.692505	4.63808	0.023484
LONRF2	-0.69313	5.10195	0.018403
LOX	-1.24735	6.5858	0.007004
LPAR5	0.787106	6.33496	0.030928
LRFN4	-0.6741	5.97566	0.029617
LRIG3	-0.67088	11.0084	0.032896
LRMP	1.46136	5.1272	0.028545
LRP5	-0.84228	6.92666	0.008314
LRRC16A	-0.69714	6.76403	0.049277
LRRC17	-1.15096	6.2246	0.000363
LRRC3	0.743811	6.16834	0.025944
LRRC8C	0.925767	5.46354	0.033387
LRRN4CL	-0.94272	5.80961	0.007064
LST1	-0.60948	4.78551	0.005783
LTB	-1.08622	8.3755	0.044487
LY86	0.665363	7.1797	0.029464
LYST	-1.02791	8.26894	0.001556
LYZ	0.846157	4.39823	0.030882
MAFB	1.14305	8.68344	0.020794
MAGI2	-0.60748	5.29377	0.010645
MAN1A1	-0.63717	4.89025	0.015875
MAP4K1	-0.97517	3.9783	0.002005
MAPK12	-0.62196	6.91481	0.026972
MAPK15	1.15472	6.25001	0.021191
MATN2	-0.61559	4.39252	0.006924
MCM10	0.880602	6.9871	0.047312
METRNL	-0.75117	5.75451	0.005864
METTL7A	-1.58586	7.1798	0.000714
MINPP1	-1.21369	7.05701	0.001881
MPZL1	1.50765	6.13968	0.015288
MR1	-0.88995	5.72764	0.035601
MRGPRX4	-0.88654	5.06053	0.012439
MRPS2	-0.99775	4.7584	0.030869
MRPS33	-0.93766	11.8717	0.015616
MS4A6A	-0.6488	4.78367	0.012
MS4A7	-0.69166	8.09302	0.042021
MSI2	-0.94639	10.8713	0.01034
MSRA	0.622246	9.81797	0.03937
MTP18	-1.07698	7.33339	0.001022
MUM1L1	-0.807	5.11611	0.034413
MYLIP	1.36755	10.2217	0.000662
N4BP2L1	1.00701	8.11987	0.027379

NACC2	-0.59091	8.45487	0.02963
NAPSB	-0.8113	3.39397	0.012791
NCF1	-1.08941	7.01284	0.007121
NCF1C	-0.69657	7.43573	0.034234
NCF2	-0.98242	11.7994	0.026778
NCKAP1L	-1.49318	6.93274	0.003433
NCRNA00219	0.690131	5.61665	0.049393
NDRG1	0.662688	6.7668	0.029691
NDUFA5	-0.98071	9.23569	0.019841
NDUFB2	-0.69657	7.43573	0.034234
NECAB2	-1.15349	5.92473	0.001762
NEFL	0.743694	7.29644	0.001299
NELL2	0.938585	9.3979	0.013223
NGF	-0.78607	10.1252	0.011083
NKG7	-0.82131	6.89381	0.004915
NLGN4X	-0.67152	3.6614	0.013634
NLRC5	-0.79668	5.10153	0.011104
NME5	-0.98614	4.2329	0.02375
NMU	0.845635	4.37593	0.035427
NOD2	0.58874	4.75598	0.020671
NOL9	0.703848	6.65975	0.020257
NOLC1	-1.53776	8.97448	0.037771
NPAS1	0.816304	6.25632	0.016969
NR4A2	0.692505	4.63808	0.023484
NTSR1	0.705342	7.64007	0.023151
NUPR1	-0.75556	6.29134	0.046664
OAS1	-1.4906	5.97139	0.001204
OAS2	1.18747	8.96259	0.003352
OAS3	0.610101	8.05098	0.016579
ODC1	0.770445	4.3042	0.017301
OLR1	0.811288	6.33933	0.01259
OSBPL10	-0.79598	6.28799	0.002787
P2RY8	ILMN_1688775	0.854399	2.44693
P4HA2	0.676854	5.392	0.02639
PAEP	-0.74634	5.76975	0.017279
PAPSS2	0.966738	6.30247	0.046793
PARP14	0.5909	11.1228	0.006569
PARP9	-0.64435	6.6478	0.014035
PARVG	-1.24735	6.5858	0.007004
PCDHB13	0.854399	7.25797	0.021154
PCDHB16	-0.71859	6.67843	0.0217
PCDHB4	-0.76965	4.7908	0.01256
PDIA6	-1.02895	6.09587	0.004813

PER3	-1.17355	6.1578	0.001403
PFKFB3	-1.17355	6.1578	0.001403
PFKFB4	-0.96746	4.97602	0.000411
PFKP	-0.78627	7.03471	0.028838
PGK1	-0.62461	4.19736	0.018616
PHKA1	-0.89541	9.15631	0.029949
PIK3AP1	-1.15096	6.2246	0.000363
PITPNC1	1.08019	6.39055	0.003059
PKD2	0.754631	4.95037	0.02931
PKMYT1	-0.72362	7.31963	0.021001
PLAC8	-1.40979	6.75682	0.000377
PLCB2	-1.26629	5.56524	0.012934
PLEK	0.632261	6.07111	0.02628
PLEKHG2	-1.30328	7.72784	0.000919
PLOD1	0.768769	5.55322	0.00528
PLXDC2	0.592449	8.13695	0.018565
PLXNA2	-1.02895	6.09587	0.004813
PLXNB1	-0.63717	4.89025	0.015875
PLXNC1	0.599159	3.96246	0.034413
PNMAL1	0.722922	5.71202	0.049822
PPME1	0.755085	7.06778	0.014272
PPP1R16B	0.738903	7.31336	0.013443
PREX1	-0.82297	6.15398	0.022478
PRKCB	-0.84085	4.45933	0.047954
PRND	-0.73444	7.69532	0.005285
PROX1	0.599232	8.80695	0.018535
PRR11	0.692505	4.63808	0.023484
PRSS35	-0.60008	5.39659	0.021565
PSMB8	-0.74149	5.46264	0.017074
PSMB9	-1.00543	9.70931	0.027283
PSTPIP1	-0.8835	10.3061	0.008569
PTGS2	-0.9437	9.1805	0.004518
PTHLH	-1.07423	5.77787	0.024086
PTPN3	1.32235	5.69683	0.01138
PTPRCAP	-1.3656	8.58106	0.003172
PTPRO	-0.68848	4.91787	0.011828
PVRIG	-0.60948	4.78551	0.005783
PVT1	0.73937	7.60201	0.014359
PYGB	0.700834	4.69033	0.023103
PYHIN1	-0.86961	7.95987	0.002622
RALGPS1	-1.13634	8.92196	0.003419
RARRES1	-1.12101	7.57496	0.004552
RARRES3	1.17349	6.50387	0.008137

RASAL3	-1.28888	6.38788	0.025963
RASGRP1	0.824532	7.13253	0.021349
RBP7	0.933129	8.39379	0.010892
RCBTB2	0.878292	8.35657	0.021702
RCSD1	1.08019	4.82271	0.017759
RFC3	-1.03582	10.5563	0.001102
RGS18	0.661092	12.4184	0.033962
RGS22	1.2497	7.96568	0.002946
RIPK3	-0.63818	5.50205	0.020672
RNASE6	0.695476	7.31094	0.023372
RNASEL	1.34353	4.98175	0.003101
ROPN1	-0.92745	5.72927	0.002566
RPE65	0.644833	6.09392	0.005364
RPS7	-0.69313	5.10195	0.018403
RRAS2	0.831194	4.78843	0.002358
RSAD2	-0.74652	4.48095	0.00094
RTP4	0.779539	6.52256	0.03727
SAMD9	1.16152	4.95368	0.030548
SAMD9L	-0.81313	5.70544	0.027371
SAMSN1	-0.73444	7.69532	0.005285
SAP18	-0.92537	8.64043	0.018536
SASH3	-0.69065	5.27928	0.007228
SC65	-0.77295	5.5038	0.000616
SCUBE3	0.693946	7.08812	0.001299
SELL	-0.65857	8.60681	0.013034
SEMA4A	-0.84228	6.92666	0.008314
SEMA4D	0.856266	8.77895	0.005448
SERINC2	-1.1746	6.4556	0.034128
SERP2	-0.87639	6.86183	0.007054
SERPINE1	-0.77662	10.9647	0.016321
SERPINH1	-0.63148	5.12001	0.018441
SERPINI1	1.15478	3.69765	0.030655
SH3PXD2A	-0.83162	5.64979	0.016191
SHB	-0.69845	5.2257	0.014147
SIDT2	-0.69673	6.81579	0.025013
SIGMAR1	-1.07109	6.57351	0.011522
SIK1	0.926311	7.56538	0.011496
SIRT6	-1.07705	7.29754	0.000415
SKA1	-0.71859	6.67843	0.0217
SLAMF1	1.1097	4.84815	0.021644
SLAMF6	-1.2507	6.70385	0.020015
SLAMF9	0.622246	9.81797	0.03937
SLC12A8	-0.86948	8.21581	0.020485

SLC15A3	-0.91609	4.54402	0.033114
SLC17A9	-1.07785	6.39801	0.02395
SLC24A3	1.36639	8.2954	0.001792
SLC25A22	-1.74116	8.74859	0.005695
SLC2A1	-0.76461	11.7131	0.001209
SLC30A1	-0.77662	10.9647	0.016321
SLC37A3	0.644833	6.09392	0.005364
SLC40A1	-0.75973	8.5482	0.020476
SLC6A15	0.743694	7.29644	0.001299
SLC7A7	0.877391	5.96314	0.029847
SLFN12	0.879717	7.35813	0.013911
SNORD1C	-0.66597	6.84528	0.049395
SOX8	-1.05786	6.4062	0.024514
SP110	-1.08812	5.06233	0.003245
SPIB	-0.79616	5.85798	0.034547
SPOCK2	-1.35108	6.01275	0.000855
SPRR2D	-0.63617	5.70956	0.016868
SPRR2F	-0.64434	3.01807	0.003469
SQRDL	-1.21648	5.00784	0.002287
ST3GAL1	-0.76492	10.1206	0.005013
STAP2	1.02622	9.51626	0.000907
STAT1	-0.62483	6.04644	0.006275
STAT2	-1.03174	8.18173	0.006455
STAT4	-0.86803	6.80365	0.023915
SYK	-0.85055	6.2078	0.031867
SYNPR	0.73937	7.60201	0.014359
TAGLN3	-0.61797	5.59769	0.007245
TAP1	-0.63148	5.12001	0.018441
TAP2	-0.98071	9.23569	0.019841
TBC1D10C	1.66981	4.358	0.01003
TBX3	-0.73584	9.67758	0.008026
TFPI2	-0.64265	4.88066	0.032344
THAP4	1.61733	4.71375	0.03771
THBS4	-0.64141	11.8135	0.014514
TK1	-1.07384	5.10776	0.02908
TLR1	1.76535	4.65324	0.014017
TLR4	-1.11985	11.2701	0.008771
TLR7	-1.02937	7.95312	0.004536
TM4SF19	-0.92745	5.72927	0.002566
TMC7	-0.93723	5.41256	0.006062
TMEM108	-0.79209	4.92244	0.002804
TMEM140	-0.94942	8.02858	0.014809
TMEM170B	1.42004	9.15518	0.048041

TMEM173	-0.60354	4.55826	0.034794
TMEM20	-1.05257	7.69379	0.006354
TMEM48	1.50356	5.44486	0.018138
TMEM84	-1.53139	7.46881	0.001923
TMEM87B	-1.02895	6.09587	0.004813
TMOD1	-0.7653	6.35834	0.014963
TMOD2	-1.09111	6.2764	0.000272
TNF	0.698046	6.00347	0.013853
TNKS1BP1	-0.60468	7.35188	0.006421
TNS3	-0.89068	7.38677	0.002446
TP53TG1	0.647168	5.45141	0.048019
TRAM1L1	-0.84674	7.42076	0.003654
TRAP1	1.18704	9.43782	0.007781
TREM1	1.04613	6.51132	0.005696
TRIM21	-0.58567	3.20601	0.004617
TRIM22	-0.63117	6.95954	0.023159
TRIM6	-1.32214	5.9152	0.000546
TRIML2	-0.84437	6.32564	0.00226
TRIP13	-0.61268	5.5406	0.026628
TSKU	-0.78468	7.68263	0.028019
TSPAN13	1.27898	6.21156	0.002846
TSPAN32	0.914716	5.97495	0.011563
TTC26	-1.14434	11.123	0.004302
TTLL12	-1.02791	8.26894	0.001556
TUBB2C	1.19507	4.56746	0.023396
TWIST2	1.06621	6.47437	0.004489
TXNIP	0.663911	8.12403	0.007123
TYROBP	0.705342	7.64007	0.023151
UBA7	-0.70305	4.59628	0.025554
UBD	-0.75779	7.46466	0.004753
UBE2L6	-0.8835	10.3061	0.008569
UBQLNL	0.856899	5.85922	0.017505
UCN2	-1.09423	7.62953	0.000091
USP51	-0.71127	6.69197	0.015571
VANGL2	0.840975	4.34693	0.010883
VAT1L	-0.85246	6.01875	0.017972
VAV1	-0.727	10.5794	0.011407
VEGFA	1.29438	6.94671	0.006613
VEGFC	-0.82111	5.76685	0.007946
VGF	-0.74967	6.10358	0.019496
VILL	-0.69176	5.11942	0.023106
WAS	1.23223	5.76574	0.019925
WASF3	-0.89541	9.15631	0.029949

WFDC1	-1.20244	4.43726	0.001402
WISP2	-0.89541	9.15631	0.029949
WT1	-1.09924	5.46827	0.028094
ZBED2	-1.04208	6.65944	0.000028
ZBP1	-0.66136	5.61421	0.020845
ZFP14	-0.80871	6.48139	0.020719
ZFP30	-0.85466	5.03546	0.016062
ZMYND15	-0.85095	6.45782	0.023095
ZNF10	-0.63818	5.50205	0.020672
ZNF185	-0.59841	6.85835	0.02826
ZNF222	-0.98374	8.85453	0.012805
ZNF226	0.5909	11.1228	0.006569
ZNF233	-0.72416	4.86992	0.010187
ZNF256	-0.7513	6.79692	0.019377
ZNF329	-1.09758	9.97148	0.006008
ZNF347	-1.03126	7.77924	0.02328
ZNF416	0.854399	7.25797	0.021154
ZNF442	-1.00271	6.82981	0.001843
ZNF583	-1.03538	7.20467	0.04943
ZNF585A	-0.7653	6.35834	0.014963
ZNF683	-0.82018	11.1146	0.004677
ZNF696	-1.15771	7.16379	0.01027
ZNF816A	0.737	6.77388	0.013972
ZNF83	-0.67659	5.599	0.038568
ZNF85	-1.01481	6.93932	0.017161
ZSCAN16	0.662688	6.7668	0.029691
